# Impact of Blood Parameters and Normal Tissue Dose on Treatment Outcome in Esophageal Cancer Patients Undergoing Neoadjuvant Radiochemotherapy

**DOI:** 10.3390/cancers14143504

**Published:** 2022-07-19

**Authors:** Rebecca Bütof, Laura Häberlein, Christina Jentsch, Jörg Kotzerke, Fabian Lohaus, Sebastian Makocki, Chiara Valentini, Jürgen Weitz, Steffen Löck, Esther G. C. Troost

**Affiliations:** 1 OncoRay—National Center for Radiation Research in Oncology, Faculty of Medicine and University Hospital Carl Gustav Carus, Technische Universität Dresden, Helmholtz-Zentrum Dresden-Rossendorf, 01307 Dresden, Germany; laura.haeberlein@uniklinikum-dresden.de (L.H.); christina.jentsch@uniklinikum-dresden.de (C.J.); fabian.lohaus@uniklinikum-dresden.de (F.L.); sebastian.makocki@uniklinikum-dresden.de (S.M.); chiara.valentini@uniklinikum-dresden.de (C.V.); steffen.loeck@oncoray.de (S.L.); esther.troost@uniklinikum-dresden.de (E.G.C.T.); 2 Department of Radiotherapy and Radiation Oncology, Faculty of Medicine and University Hospital Carl Gustav Carus, Technische Universität Dresden, 01307 Dresden, Germany; 3 National Center for Tumor Diseases (NCT), Partner Site Dresden, Germany: German Cancer Research Center (DKFZ), Heidelberg, Germany; Faculty of Medicine and University Hospital Carl Gustav Carus, Technische Universität Dresden, Dresden, Germany, and Helmholtz Association/Helmholtz-Zentrum Dresden-Rossendorf (HZDR), 01307 Dresden, Germany; joerg.kotzerke@uniklinikum-dresden.de (J.K.); juergen.weitz@uniklinikum-dresden.de (J.W.); 4 Helmholtz-Zentrum Dresden-Rossendorf, Institute of Radiooncology—OncoRay, 01328 Dresden, Germany; 5 Department of Nuclear Medicine, Faculty of Medicine and University Hospital Carl Gustav Carus, Technische Universität Dresden, 01307 Dresden, Germany; 6 Department of Visceral, Thoracic and Vascular Surgery, Faculty of Medicine and University Hospital Carl Gustav Carus, Technische Universität Dresden, 01307 Dresden, Germany; 7 German Cancer Consortium (DKTK), Partner Site Dresden, and German Cancer Research Center (DKFZ), 69192 Heidelberg, Germany

**Keywords:** esophageal cancer, radiochemotherapy, normal tissue dose, neoadjuvant, outcome, PET, blood parameters

## Abstract

**Simple Summary:**

Prognosis of patients with locally advanced esophageal tumors remains poor despite ongoing efforts to improve treatment options. Nevertheless, neoadjuvant radiochemotherapy (RCTx) followed by surgery has resulted in increased survival rates. One critical point for irradiation of esophageal cancer is the optimal sparing of surrounding normal tissue, especially for the lungs, heart and bone marrow, in the immediate neighborhood of the target volume, since corresponding toxicity could impact the outcome of treated patients. Therefore, the aim of our study was to assess the potential influence of normal tissue radiation dose and blood parameters on survival in this patient cohort. Furthermore, functional imaging parameters of these organs at risk, extracted from 18F-FDG-PET/CT before and during neoadjuvant RCTx, have been correlated with the radiation dose to normal tissues and the blood parameters of interest. We found a significant association of higher radiation doses to the lungs and heart with overall survival. In contrast, neither functional imaging parameters nor blood values were prognostic in this neoadjuvant patient cohort.

**Abstract:**

Despite technological advances, normal tissue sparing in photon beam irradiation is still challenging. Since in esophageal cancer this may inflict damage on the lungs, heart and bone marrow, possibly impacting on outcome, the aim of this study was to investigate the association of normal tissue dose and blood parameters on the survival of patients having undergone neoadjuvant radiochemotherapy (RCTx) followed by surgery. This retrospective study included 125 patients irradiated to 40–41.4 Gy with photons or protons combined with concurrent chemotherapy. On initial and restaging 18F-FDG-PET/CT, the lungs and heart were contoured as organs at risk for which standardized uptake values (SUV) were evaluated. The mean radiation dose (D_mean_) to the lungs and heart, the volume of the lungs receiving at least 20 Gy (V_20Gy_lung_) and various pre- and per-treatment blood parameters were included in the Cox regression analyses. Results: The median follow-up time was 19.8 months and median overall survival 37 months (95% confidence interval: 16–58.9 months). In multivariate analysis, higher radiation doses to the lungs and heart were statistically significantly associated with decreased overall survival (D_mean_lung_: *p* < 0.001; V_20Gy_lung_: *p* < 0.002; D_mean_heart_: *p* = 0.005). Neither the 18F-FDG-PET nor blood parameters were predictive for overall survival. In patients with locally advanced esophageal cancer treated with RCTx, the radiation dose to the heart and lungs was significantly associated with overall survival.

## 1. Introduction

Despite ongoing efforts to increase the effectiveness of treatment options, the prognosis for patients with esophageal carcinoma remains poor, with an average overall survival of 15% after 5 years [1]. Patients with locally advanced esophageal cancer are treated with neoadjuvant radiochemotherapy (RCTx) and resection since several randomized trials have shown the benefit of this combined treatment approach instead of surgery alone [2,3,4]. Although this improvement is encouraging, clinical data suggest heterogeneity of responses in individual patients. Several clinical and imaging parameters have already been investigated in order to predict therapy outcome in this patient cohort [5,6,7]. Based on results for (non-)small cell lung cancer patients, the impact of irradiated normal tissue on survival rates has become a new focus of research [8,9]. It has been shown that, in particular, the radiation dose to the lungs and heart significantly influences treatment outcomes due to induced morbidity and mortality. The major reason is that, even though technological advances in radiotherapy have been implemented recently, normal tissue sparing during (photon beam) irradiation is still challenging since radiation-sensitive organs at risk are in direct proximity. Therefore, the first aim of this study was to investigate the association of radiation doses applied to the lungs and heart with survival in patients having undergone neoadjuvant RCTx.

In addition to the dose to organs at risk, recently published studies have revealed a significant association of radiation dose applied to circulating immune cells and RCTx-induced lymphopenia with worsened clinical outcome in esophageal cancer patients [10,11]. This recent aspect has not been implemented in clinical routine so far. For individualization of treatment concepts, a valid prognostic and predictive model is needed. In order to improve prediction of the outcome in esophageal cancer patients receiving RCTx, a combination of quantitative assessment of functional tumor volume information provided by [18F]-fluorodeoxyglucose positron emission tomography (FDG PET) with proven basic clinical parameters has been shown to be promising [5,12]. Furthermore, FDG uptake in irradiated normal esophageal tissue measured on restaging PET also has significant prognostic value in this patient cohort [13].

To bridge the gap of functional imaging to the recently found blood parameters of interest, the second aim of the study was to investigate whether the absolute values of blood parameters (hemoglobin value (Hb), platelet and leukocyte counts) collected before and during RCTx, as well as their per-treatment changes, correlated with PET data or with normal tissue dose to the heart and lungs. Moreover, the functional imaging parameters were correlated with the radiation dose to normal tissues.

## 2. Materials and Methods

### 2.1. Patient Characteristics

In the present study, 125 consecutive patients with 18F-FDG PET/CT-staged esophageal carcinoma who received neoadjuvant RCTx with curative intent between January 2010 and January 2019 were retrospectively analyzed. The study was approved by the Ethics Committee of the Technische Universität Dresden, and all subjects provided written informed consent to use their data for scientific purposes.

Inclusion criteria were: ≥18 years, good performance status, histologically confirmed esophageal adenocarcinoma or squamous cell carcinoma, no distant metastases, neoadjuvant RCTx with curative intent, initial 18F-FDG PET/CT before treatment as well as in the last week of RCTx and corresponding blood samples at least before/after initial imaging.

### 2.2. Treatment

The patients were irradiated with photons (*n* = 116; 3D-RT or IMRT) or protons (*n* = 9; double scattering) to a total dose of 40–41.4 Gy in 20 to 23 fractions. Gross tumor volume (GTV) was defined as primary tumor and any suspect lymph nodes visualized during endoscopy and endo-esophageal ultrasound, on CT (short axis of lymph nodes >1 cm) or with 18F-FDG-PET avidity. The clinical target volume (CTV) was obtained by expanding the GTV using a margin of 1.5 cm circumferentially and 2–3 cm cranio-caudally for primary tumor, and 1.0 cm for lymph nodes, subsequently corrected for anatomical boundaries. From January 2010 to January 2017, elective lymph node stations (ENI) were irradiated in addition (*n* = 98). The clinical target volume was expanded to a planning target volume using institutional margins of 0.8 cm for photons. Proton treatment planning was based on the CTV; range and setup uncertainties were taken into account during planning (aperture/compensator).

The first cycle of guideline-based concurrent chemotherapy (weekly carboplatin AUC2 and paclitaxel 50 mg/m^2^ or 5-FU combined with cisplatin in weeks 1 and 4) started in the first week of irradiation. All patients had surgery planned after neoadjuvant RCTx.

### 2.3. FDG PET Protocol and Data Analysis

All patients underwent a hybrid 18F-FDG PET/CT scan prior to treatment. A second scan was performed during the last week of RCTx. Scans (3D PET acquisition, 3 min emission per bed position) were performed on a Biograph 16 (Siemens Medical Solutions Inc., Knoxville, TN, USA). Data acquisition started, on average, 60 min after injection of 310 to 360 MBq 18F-FDG taking into account patient weight. All patients had fasted for at least 6 h prior to tracer injection.

**Figure 1:**
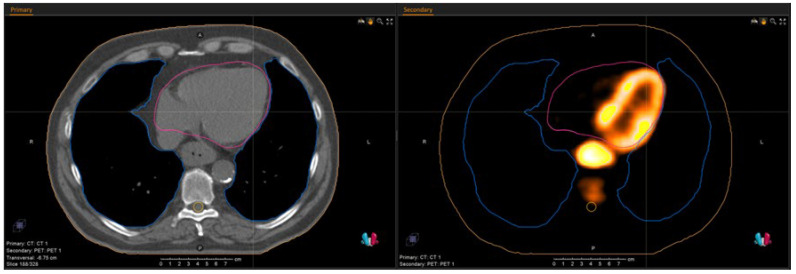
Transversal layer of pretherapeutic CT (left) and 18F-FDG-PET (right); blue: lung contour; pink: heart contour.

Image analyses and extraction of radiation doses from the clinically applied treatment plans were performed using the RayStation Research software, version 8.1 (RaySearch Laboratories AB, Stockholm, Sweden). On 18F-FDG-PET/CT, acquired for treatment planning and restaging, respectively, the lungs and heart were contoured as a region of interest (ROI) by one experienced radiation oncologist according to published guidelines [14]. From the radiation treatment plans, the mean dose (D_mean_) to the lungs and heart as well as the volume of the lungs receiving more than 20 Gy (V_20Gy___lung_) were extracted. For the delineated ROIs (see Figure 1), maximum and mean standardized uptake values (SUV_max_, SUV_mean_) were computed. Additionally, the relative difference from first to second PET scan (reference: first scan) for these parameters (∆SUV_max_, ∆SUV_mean_) of the lung and heart was used for statistical analyses.

Data of blood samples (hemoglobin value (Hb), platelet, leukocyte, lymphocyte, neutrophilic granulocyte and monocyte counts) 7 days before/after initial 18F-FDG PET and before/after restaging imaging in the 4th week of treatment were collected in all available cases. The actual values and the differences between the two time points were analyzed. Furthermore, surgical complications were recorded for analysis of their impact on survival rates.

### 2.4. Clinical Endpoints and Statistical Analysis

The three clinical endpoints of this study were overall survival (OS), local tumor control (LC) and freedom from distant metastases (FFDM), measured from the start of radiotherapy until event or last follow-up in case of censoring.

The software Excel 2019 (Microsoft, Redmond, WA, USA) was used for pseudonymized data collection, and all statistical analyses were performed using SPSS version 27 (IBM Corporation, Armonk, NY, USA).

The prognostic value of clinical factors (TNM status, age, surgical complications), normal tissue dose parameters for lungs (D_mean_lung_, V_20Gy_lung_) and heart (D_mean_heart_), PET values (SUV_max_, SUV_mean_, ∆SUV_max_, ∆SUV_mean_) and blood parameters on OS, LC and FFDM was tested using univariate Cox regression, with *p*-values ≤ 0.05 considered statistically significant. Thereafter, prognostic independence was analyzed in multivariate Cox regression including all significant parameters according to univariate evaluation. Referring to [15], the respective cut-off values for significant DVH parameters were determined for overall survival based on the maximal Youden index of the receiver operating characteristics (ROC) curve of the binary endpoint.

Spearman’s correlation analyses were performed to investigate the correlation between the absolute values of blood parameters (available for all patients) collected before and during RCTx, their per-treatment changes and PET parameters or dose to the heart and lungs. Since, e.g., lymphopenia is a known prognostic factor associated with applied body/vertebral irradiation dose [16], we hypothesized further associations with the dose to organs at risk/imaging changes, in particular, at the time of restaging. Possible correlations between functional imaging parameters and radiation dose to normal tissue were also evaluated by Spearman’s correlation coefficient.

## 3. Results

The median age of all included patients was 61 years (range 40 to 79), and the majority were male (90%). The sites of tumor locations were classified as follows: upper part of the esophagus (*n* = 2), middle part (*n* = 45), lower part (*n* = 78). A summary of patient and tumor characteristics is given in Table 1. Eleven patients (nine percent) did not receive the initially planned chemotherapy schedule as a result of toxicities (e.g., febrile infections). Moreover, in nine patients, surgical tumor resection was impossible due to the metastatic situation after restaging or bad general condition, and in nine additional cases, the surgical resection had to be discontinued due to different reasons (e.g., aortic infiltration or cardiac arrhythmias).

The 2-year, 3-year and 5-year overall survival rates were 57.8%, 51.2% and 42.7%, respectively. The median overall survival was 37 months (95% confidence interval: 16.0–58.9). During the follow-up period, 35 patients died from esophageal cancer, and 27 died of non-tumor-related causes. The DVH parameters did not significantly differ between patients with different causes of death (D_mean_lung_: *p* = 0.97, V_20Gy_lung_: *p* = 0.94, D_mean_heart_: *p* = 0.36), i.e., they showed a similar effect on overall survival for both groups.

In our study, local tumor control was 67.9%, and freedom from distant metastases was 44.4% after five years. Median local control was not reached during follow-up, while median freedom from distant metastases was 44.4 months (95% confidence interval: 20.2–68.5).

An overview of all investigated blood parameters, of the PET values at the time of staging and restaging, and of the applied normal tissue doses is shown in Table 2. D_mean_lung_, V_20Gy_lung_ and D_mean_heart_ were significantly higher in patients receiving elective nodal irradiation compared to patients without ENI (*p* < 0.001).

### 3.1. Prognostic Factors for Overall Survival

According to univariate Cox regression analyses (see Table 3), higher normal tissue doses to the lungs and heart were significantly associated with decreased overall survival (D_mean_lung_: *p* < 0.001; V_20Gy_lung_: *p* < 0.001; D_mean_heart_: *p* = 0.002). Furthermore, the occurrence of complications after surgical tumor resection significantly worsened overall survival (*p* = 0.047). The most common surgical complication, anastomotic leakage, was predictive of overall survival (*p*-value = 0.010). In addition, overall survival was significantly associated with clinical N-stage of the patients (overall *p* = 0.012; cN0 vs. cN1 *p* = 0.015 and cN0 vs. > cN0 *p* = 0.025).

Neither baseline or restaging blood parameters available for all patients nor 18F-FDG-PET parameters were prognostic for overall survival. Blood counts of lymphocytes, monocytes and neutrophilic granulocytes were only available in 45 patients at staging and in 26 cases at restaging. For overall survival, restaging lymphocyte values (in favor of lower counts; *p* = 0.014) and lower initial monocyte counts (*p* = 0.005) showed significant prognostic associations.

The significant dose parameters were individually combined with cN-stage, anastomotic leakage, age and histology in multivariate Cox regression analyses. A lower radiation dose applied to the lungs (D_mean_lung_: *p* = 0.001, V_20Gy_lung_: *p* = 0.002) and to the heart (D_mean_heart_: *p* = 0.005) were found to be significant prognostic factors for longer overall survival. In addition, all three analyses revealed that the occurrence of an anastomotic leakage was significantly associated with worse overall survival.

**Figure 2:**
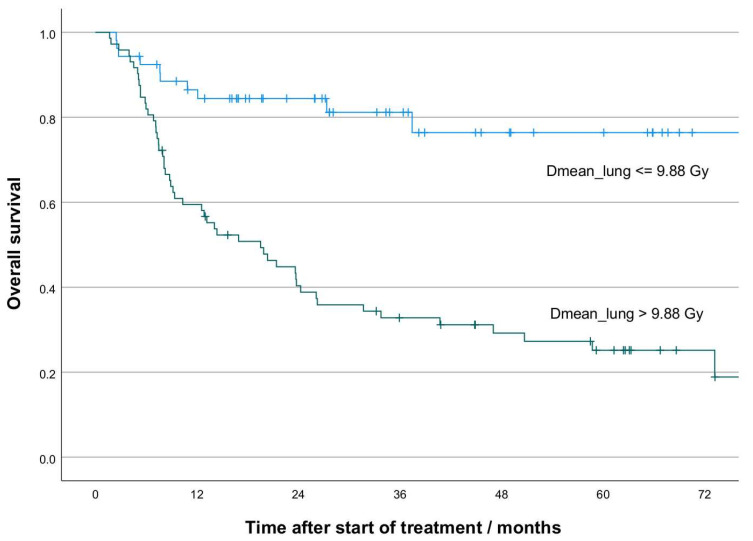
Exemplary Kaplan–Meier curves for overall survival based on the stratification by D_mean_lung_ (*p* < 0.001). Optimal cut-off values for respective DVH parameters have been determined based on the maximal Youden index of the receiver operating characteristics (ROC) curve of the binary endpoint.

The optimal cut-off values for all three DVH parameters regarding overall survival in our cohort were D_mean_lung_: 9.88 Gy, (Youden Index 0.516); V_20Gy_lung_: 13.5%, (Youden Index 0.487); and D_mean_heart_: 25.8 Gy (Youden Index 0.384). All three cut-offs led to patient stratifications with a significant difference in overall survival (log-rank test: *p* < 0.001). The cut-off of 23% proposed in [15] for V_20Gy_lung_ also led to a significant patient stratification (*p* = 0.013) but with lower discrimination. As an example, the Kaplan–Meier curves based on the stratification by D_mean_lung_ are shown in Figure 2.

### 3.2. Prognostic Factors for Local Tumor Control

Univariate Cox regression revealed that a better WHO status (grade 0 vs. grade 1–2; *p* = 0.040) as well as lower D_mean_lung_ (*p* = 0.009) and V_20Gy_lung_ (*p* = 0.037) values were significantly associated with longer local tumor control. All other investigated parameters were not prognostic for local tumor control. In multivariate Cox regression, taking into account the WHO status combined with D_mean_lung_ or V_20Gy_lung_ individually, only D_mean_lung_ remained statistically significant (*p* = 0.035).

### 3.3. Prognostic Factors for Freedom from Distant Metastases

None of the clinical parameters were prognostic for freedom from distant metastases. Furthermore, none of the baseline or restaging PET and blood parameters showed a significant correlation with this clinical endpoint. The applied radiation dose to normal tissue was also not significantly associated with the occurrence of distant metastases.

### 3.4. Correlation of Dose Parameters, Blood Values and PET Data

**Figure 3:**
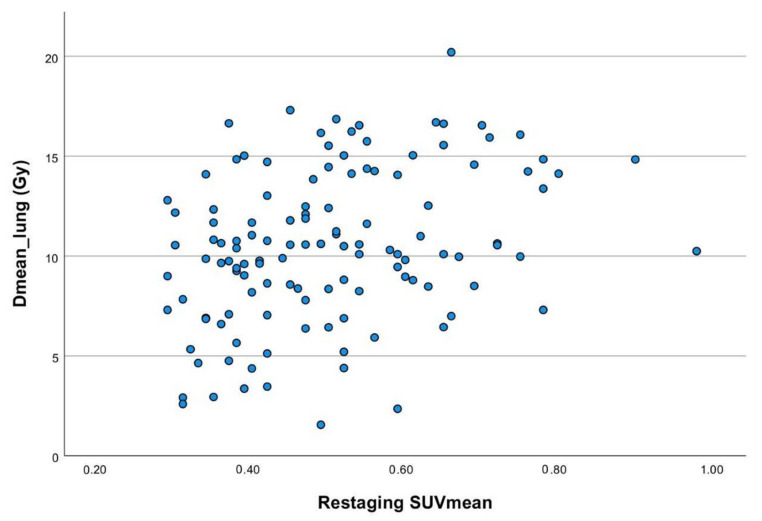
Scatter plot of applied D_mean_lung_ and corresponding SUV_mean_ values in restaging 18F-FDG-PET.

For the lungs, moderate significant correlations were observed between the applied dose and SUV in restaging 18F-FDG-PET. The mean lung dose showed a Spearman’s correlation of 0.32 with restaging SUV_mean_ (see Figure 3) and 0.29 with restaging SUV_max_, while for V_20Gy_lung_, correlations of 0.32 and 0.23 were found, respectively. For the heart, correlations between D_mean_heart_ and restaging or ∆SUV were even smaller (<0.14).

The absolute values of blood parameters collected pre- or per-RCTx did not correlate with 18F-FDG-PET, either at one of the two time points or with their changes over time. Moderate negative correlations were found between the restaging hemoglobin value and dose to the heart (−0.27) and lungs (D_mean_lung_ (−0.20) and V_20Gy_lung_ (−0.24)). Moreover, no significant correlation was found between blood value changes over time and corresponding normal tissue doses.

## 4. Discussion

In this exploratory study, we found that, upon multivariate analysis, lower radiation doses applied to the lung and to the heart were significant prognostic factors for longer overall survival after trimodal treatment for esophageal carcinoma. Furthermore, the quantitative assessment of the PET data in normal tissues did not correlate with either the absolute blood values collected before or during RCTx or with their per-treatment changes. Finally, none of the PET values or blood parameters available for all patients were predictive for overall survival.

The impact of heart dose on outcome in patients with esophageal carcinoma receiving radiochemotherapy with definitive or neoadjuvant intent is an important topic that has recently become a new focus of research. One of the underlying reasons was the unexpected results of trial RTOG 0617 that showed decreased survival rates for patients with non-small cell lung carcinoma after dose escalation, which has been explained by an increased heart toxicity due to a higher normal tissue dose [8]. Despite late cardiotoxicity related to radiotherapy having been well-reported in other tumor entities, e.g., breast cancer or Hodgkin’s lymphoma, knowledge about cardiac dose and associated morbidity and mortality in patients with esophageal carcinoma is limited so far.

Therefore, Cai and colleagues [17] investigated heart dosimetric parameters and cardiac events in a retrospective study on esophageal patients with definitive radiotherapy. They found a significant association between higher values of mean heart dose and heart volume receiving ≥5 Gy with an increased cardiac event rate and worse overall survival [17]. Another cohort analysis reported incidence and onset of severe cardiac events in patients with esophageal carcinoma treated with either intensity-modulated photon irradiation or proton beam therapy [18]. In 18% of patients, at a median of 7 months, severe cardiac events occurred, and the mean heart dose, particularly of less than 15 Gy, was significantly associated with decreased rates of side effects [18]. In agreement with our results, this was associated with worse overall survival.

Recently published data suggested specific heart substructures as important areas of risk. Wang and colleagues [19] evaluated radiation dosimetric determinants of major coronary events and their impact on patient prognosis in esophageal patients treated with concurrent RCTx. They found the radiation dose to coronary substructures may be better predictors of toxicity than mean heart dose or whole-heart volume receiving ≥30 Gy. If these data were validated in independent patient cohorts, it would have important implications for radiation treatment planning through new definitions of organs or regions at risk. Here, proton therapy could play an important new role, since normal tissue sparing is superior to conventional radiation technology due to its physical properties, and the targeted sparing of certain cardiac substructures seems feasible [20,21]. In this regard, ongoing prospective comparative studies on photon vs. proton irradiation of thoracic tumors will provide answers for optimal therapeutic approaches. One example is the EU-funded PROTECT trial, an international multicenter clinical study randomizing photons versus protons in the trimodality treatment of esophageal carcinoma, which will start recruitment soon (ClinicalTrials.gov Identifier: NCT05055648).

The significant association of lung dose and overall survival in our study is in line with recently published data in other cohorts of esophageal cancer patients receiving RCTx. In one study with trimodality treatment, multivariate analyses revealed lung V_20Gy_ and lung V_5Gy_ to be significantly associated with overall survival [15]. Interestingly, the mean heart dose showed no association with survival outcomes in this evaluation [15]. As we also reported, other data confirm heart and lung doses as independent predictors of overall survival in esophageal cancer patients after RCTx [22]. In this study, heart V_30Gy_ > 45% and mean lung dose > 10 Gy were found to be independently associated with worse survival after adjustment for other clinical and dosimetric factors, which further underlines the necessity of the optimal minimization of normal tissue doses [22]. Especially for esophageal carcinoma, radiation planning is challenging because of the mutual influence of heart and lung doses. The results of a related investigation suggest that reducing the cardiac dose at the expense of the dose to the lungs is deemed not optimal in esophageal cancer patients [23].

For clinical routine, in addition to dose parameters alone, further prognostic factors should be taken into account. Recently published studies have revealed a significant association of RCTx-induced lymphopenia with decreased clinical outcome in esophageal cancer patients [10,11] but also in other intrathoracic tumor entities [24]. In contrast to these results, in our study, lower counts of lymphocytes at the restaging time point were significantly correlated with better overall survival. Nevertheless, our data have a major shortcoming because lymphocyte values were only available for 26 patients at restaging, which clearly limits statistical power.

Irradiation volume itself could also have an impact on certain blood values and dosimetric parameters. As already known for other tumor entities, a negative impact of GTV on prognosis has also been published for esophageal cancer patients [25,26]. The use of elective nodal irradiation (ENI) vs. selective nodal irradiation (SNI) is still controversial for esophageal cancer, and different clinical practices exist. In our cohort, all dose parameters associated with overall survival were significantly higher in patients receiving elective nodal irradiation compared to patients without ENI, which could be interpreted as a surrogate for an impact of CTV on outcome in esophageal cancer patients. This phenomenon has also been reported before, showing significant impact on toxicity rates. A retrospective analysis regarding the feasibility of involved-field irradiation resulted in decreased irradiation toxicities without reduced overall survival in elderly patients with esophageal carcinoma [27]. A meta-analysis comparison of ENI and involved-field irradiation in esophageal squamous cell carcinoma revealed lower incidences of severe radiation esophagitis and pneumonia in the latter group without significant differences in local tumor control rates or overall survival [28]. Further data also suggest that involved-field irradiation is feasible in esophageal cancer patients [29]. This could be one important aspect of decreasing normal tissue irradiation doses.

Our univariate Cox regression revealed better WHO status as well as lower D_mean_lung_ to be significantly associated with longer local tumor control. On the one hand, the WHO status could be a surrogate parameter for a complete resection (since, in some cases, surgery had to be discontinued because of, e.g., cardiac arrhythmias in patients with co-morbidities), and on the other hand, lower mean lung doses seem to correlate with smaller irradiation volumes, which could also explain better local tumor control rates. In contrast, for freedom from distant metastases, none of the investigated clinical or imaging parameter showed a significant correlation, which could be explained by the comparably narrow range of tumor burden since the majority of patients had T3 and N1 tumors, leading to a homogeneous cohort for risk of distant metastases.

To bridge the gap to the recently found blood parameters of interest, the aim and the novelty of the present study were to investigate whether the absolute values of blood parameters collected before and during RCTx and their changes over time correlate with PET data or with normal tissue dose to the heart and lungs. Since functional imaging data of tumor volume have been shown to significantly correlate with outcome in esophageal cancer patients [5,30,31,32], additional PET parameters could possibly increase predictive value. First, promising results have been published for the evaluation of a non-tumor-affected esophagus: Restaging SUV_max_ and SUV_mean_ were prognostic factors for histopathological complete response after surgery and for overall and disease-free survival in esophageal cancer patients after trimodal treatment [13]. Therefore, our question was whether PET parameters of additional organs at risk could also be used to predict tumor response since this aspect had never been investigated before. For the lungs, moderate significant correlations were observed between the applied dose and SUV in the restaging 18F-FDG-PET. For the heart, correlations between the mean heart dose and restaging or ∆SUV were smaller. This means, in our study, that the prediction of normal tissue doses and corresponding damage using functional imaging was not convincing. Furthermore, absolute values of blood parameters collected before and during RCTx showed no relevant Spearman’s correlation with 18F-FDG-PET parameters, either for the two time points or for changes over time. So far, only limited data exist regarding the evaluation of functional imaging parameters and their association with normal tissue doses or blood values in esophageal cancer patients. One smaller retrospective study with 51 patients demonstrated a trend of increasing cardiac SUV changes as a function of applied heart dose with a predictive value for overall survival [33]. Another prospective study in 12 patients using single photon emission computed tomography (SPECT imaging) revealed significant correlations between radiation dose to the heart and myocardium metabolism [34]. In contrast, no statistically significant correlations could be shown for SPECT imaging one year after radiochemotherapy, suggesting other factors than radiation dose alone for impact on long-term myocardial metabolism [34]. This underlines that clinical factors, co-morbidities, applied normal tissue doses and functional imaging data should be considered in a combined manner in order to enable optimal therapeutic concepts based on prognostic scores for esophageal cancer patients.

In general, one limitation of our study is its retrospective character, i.e., our findings have to be validated in further trials with independent patient groups. Nevertheless, the study comprises a homogeneous cohort including 125 consecutive patients and, to the best of our knowledge, gives the first comprehensive overview of dosimetric parameters, blood values and possible associations with functional imaging data.

## 5. Conclusions

In patients with locally advanced esophageal cancer treated with RCTx, normal tissue dose to the heart and lungs was significantly associated with overall survival. The influence of the cardiac dose on morbidity and mortality requires further analysis in this tumor entity.

## Figures and Tables

**Table 1:** Characteristics of patient cohort (*n* = 125).

Characteristics	Median (Range)	Number of Patients	%
Age (years)	61.3 (40.5–79.3)	-	-
Body mass index (kg/m^2^)	26.3 (16.7–43.6)	-	-
Gender	Male	113	90.4
	Female	12	9.6
WHO status	0	67	53.6
	1	51	40.8
	2	2	1.6
	Unknown	5	4.0
Histology	Adenocarcinoma	43	34.4
	Squamous Cell Carcinoma	82	65.6
Clinical T-stage	2	19	15.2
	3	96	76.8
	4	7	5.6
	Unknown	3	2.4
Clinical N-stage	0	14	11.2
	1	89	71.2
	2	13	10.4
	3	2	1.6
	Unknown	7	5.6
Chemotherapy	Complete	114	91.2
	Prematurely Terminated	11	8.8

**Table 2:** Overview of the investigated parameters. As an exception, blood counts of lymphocytes, neutrophil granulocytes and monocytes (marked with an asterisk *) were only available in 45 patients at staging and in 26 cases at restaging.

Parameter	Median (Range)	Median (Range)
	Staging	Restaging
Hb (mmol/L)	8.20 (4.90–10.40)	7.50 (5.20–9.50)
Leucocytes (GPt/L)	8.38 (4.07–26.02)	3.36 (1.49–8.91)
Thrombocytes (GPt/L)	248 (98–573)	152.5 (71–360)
Lymphocytes (GPt/L) *	1.63 (0.17–3.26)	0.30 (0.12–3.11)
Neutrophil Granulocytes (GPt/L) *	5.26 (2.67–14.83)	2.14 (0.61–6.78)
Monocytes (GPt/L) *	0.73 (0.02–1.72)	0.48 (0.09–1.08)
SUV_mean_lung_	0.48 (0.30–0.95)	0.49 (0.29–0.98)
SUV_max_lung_	3.07 (1.66–11.02)	2.56 (1.39–8.72)
SUV_mean_heart_	1.58 (1.05–6.35)	1.59 (0.95–7.39)
SUV_max_heart_	4.12 (1.93–24.81)	3.83 (2.03–28.19)
D_mean_lung_ (Gy)	10.49 (1.55–20.20)	
V_20Gy_lung_ (%)	15.95 (0.00–34.74)	
D_mean_heart_ (Gy)	26.10 (0.38–42.69)	

**Table 3:** Univariate Cox regression for overall survival (*n* = 125). The significant *p*-values are printed in bold.

Parameter (Unit)	Hazard Ratio (95% Confidence Interval)	*p*-Value
Age (years)	1.03 (1.00–1.06)	0.083
Histology (squamous cell carcinoma vs. adenocarcinoma)	0.63 (0.38–1.05)	0.077
N-stage (N0 vs. > N0)	3.80 (1.19–12.18)	**0.025**
∆Hb (mmol/l)	1.14 (0.79–1.65)	0.48
∆Leukocytes (GPt/L)	1.05 (0.96–1.14)	0.28
∆Thrombocytes (GPt/L)	1.00 (0.99–1.00)	0.19
Restaging lymphocytes (GPt/L)	12.29 (1.65–91.55)	**0.014**
Surgical complication (yes/no)	1.93 (1.01–3.69)	**0.047**
D_mean_lung_ (Gy)	1.17 (1.09–1.26)	**<0.001**
V_20Gy_lung_ (%)	1.08 (1.04–1.12)	**<0.001**
D_mean_heart_ (Gy)	1.07 (1.03–1.11)	**0.002**

## Data Availability

The data presented in this study are available on request from the corresponding author.
